# Evidence-Based Knowledge Versus Negotiated Indicators for Assessment of Ecological Sustainability: The Swedish Forest Stewardship Council Standard as a Case Study

**DOI:** 10.1007/s13280-012-0377-z

**Published:** 2013-03-10

**Authors:** Per Angelstam, Jean-Michel Roberge, Robert Axelsson, Marine Elbakidze, Karl-Olof Bergman, Anders Dahlberg, Erik Degerman, Sönke Eggers, Per-Anders Esseen, Joakim Hjältén, Therese Johansson, Jörg Müller, Heidi Paltto, Tord Snäll, Ihor Soloviy, Johan Törnblom

**Affiliations:** 1 Faculty of Forest Sciences, School for Forest Management, Swedish University of Agricultural Sciences, PO Box 43, 730 91 Skinnskatteberg, Sweden; 2 Department of Wildlife, Fish, and Environmental Studies, Swedish University of Agricultural Sciences, 901 83 Umeå, Sweden; 3 Faculty of Forest Sciences, School for Forest Management, Swedish University of Agricultural Sciences, PO Box 43, 739 21 Skinnskatteberg, Sweden; 4 Department of Physics, Chemistry and Biology (IFM), Linköping University, 581 83 Linköping, Sweden; 5 Department of Forest Mycology and Plant Pathology, Swedish University of Agricultural Sciences, PO Box 7026, 750 07 Uppsala, Sweden; 6 Department of Aquatic Resources, Institute of Freshwater Research, Pappersbruksallén 22, 702 15 Örebro, Sweden; 7 Department of Ecology, Swedish University of Agricultural Sciences, PO Box 7044, 750 07 Uppsala, Sweden; 8 Department of Ecology and Environmental Sciences, Umeå University, 901 87 Umeå, Sweden; 9 National Park Bavarian Forest, Freyunger Strasse 2, 94481 Grafenau, Germany; 10 Swedish Species Information Centre, Swedish University of Agricultural Sciences, PO Box 7007, 750 07 Uppsala, Sweden; 11 Institute of Ecological Economics, Ukrainian National Forestry University, Gen. Chuprynky 103, Lviv, Ukraine

**Keywords:** Biodiversity, Monitoring, Indicators, Performance targets, Negotiation, Social learning

## Abstract

Assessing ecological sustainability involves monitoring of indicators and comparison of their states with performance targets that are deemed sustainable. First, a normative model was developed centered on evidence-based knowledge about (a) forest composition, structure, and function at multiple scales, and (b) performance targets derived by quantifying the habitat amount in naturally dynamic forests, and as required for presence of populations of specialized focal species. Second, we compared the Forest Stewardship Council (FSC) certification standards’ ecological indicators from 1998 and 2010 in Sweden to the normative model using a Specific, Measurable, Accurate, Realistic, and Timebound (SMART) indicator approach. Indicator variables and targets for riparian and aquatic ecosystems were clearly under-represented compared to terrestrial ones. FSC’s ecological indicators expanded over time from composition and structure towards function, and from finer to coarser spatial scales. However, SMART indicators were few. Moreover, they poorly reflected quantitative evidence-based knowledge, a consequence of the fact that forest certification mirrors the outcome of a complex social negotiation process.

**Electronic supplementary material:**

The online version of this article (doi:10.1007/s13280-012-0377-z) contains supplementary material, which is available to authorized users.

## Introduction

Ecological sustainability in terms of functional ecosystems (Odum 1953) is a foundation for natural capital and thus for the delivery of ecosystem services as a base for economic and social sustainability (Kumar 2010). However, the global ecological footprint on natural capital is increasing (MEA 2005; Butchart et al. 2010). Consequently, the formulation of criteria, indicators, and verifier variables to measure status and change of ecological sustainability has proliferated in many natural resource sectors (Lammerts van Bueren and Blom 1997). In contrast, norms or performance targets that allow quantitative assessment of ecological sustainability are less developed. To improve this situation, policy and evidence-based targets have been formulated for protected areas (e.g., Maltby et al. 2006; CBD 2010), emissions of pollutants based on the critical load concept (Nilsson and Grennfelt 1988), amount of food resources for selected species groups (Cury et al. 2011), and minimum habitat requirements for species (Angelstam et al. 2004; Svancara et al. 2005; Tear et al. 2005; Groffman et al. 2006).

Voluntary market-driven mechanisms such as certification (Auld et al. 2008), eco-labeling (Amacher et al. 2004), and fair trade (Renard 2003) have become widespread tools to pursue sustainability through operational management of ecosystem services. Ultimately, one aim is to contribute to the sustainable use of natural capital by formulating norms in terms of negotiated standards against which performance can be assessed. Forest certification is a good example (Gulbrandsen 2005a, b; Auld et al. 2008), the application of which is dependent on regional market characteristics and land ownership (Keskitalo et al. 2009). The Forest Stewardship Council’s (FSC) approach is one of the most widespread systems globally (Auld et al. 2008) and its application is growing (Sparks et al. 2011). The mission of FSC^1^ is ‘to promote environmentally appropriate, socially beneficial, and economically viable management of the world’s forests’. Environmentally appropriate forest management has to ‘ensure that the harvest of timber and non-timber products maintains the forest’s biodiversity, productivity, and ecological processes’. This means that FSC can be viewed as a tool that can potentially contribute to the implementation of ecological sustainability by considering evidence-based knowledge about ecosystems (see Electronic Supplementary Material, S1). FSC has created a global generic standard with principles and criteria that define what well-managed forests are. Furthermore, nationally or regionally negotiated indicators, adapting the framework of the globally valid principles and criteria, may be approved by FSC if the indicator development, negotiation, and decision-making processes follow the pre-defined guidelines (Auld et al. 2008; Elbakidze et al. 2011).

Many reports claim that FSC certification improves forest management practices worldwide (e.g., Karmann and Smith 2009). However, while researchers have analyzed the political, economic, and social outcomes of FSC (e.g., Cashore et al. 2003, 2005; Auld et al. 2008), few independent studies have addressed how FSC standards match evidence-based knowledge about requirements for ecological sustainability with different levels of ambition (Dahl 2000, 2001), or to what extent FSC certification actually contributes to ecological sustainability on the ground (Rametsteiner and Simula 2003; Gulbrandsen 2005a; Tikina and Innes 2008; Kitayama 2013). There is currently a widespread interest for independent assessment, from a wide variety of stakeholders, of FSC standard’s ecological foundation, the ecological consequences on the ground, and how to measure this. FSC members need this information at the national level both as feedback for standard revisions, and to justify customers’ and public support for FSC in general. For example, the Swedish forest industry has initiated its own assessments of ecological sustainability issues related to forestry and see the need for improvements (Skogsindustrierna 2011). Additionally, concerns have been expressed by environmental organizations about the ecological outcomes of FSC certification (Anon. 2008). As a result, some environmental non-governmental organizations have even ceased to support FSC Sweden due to the standard’s perceived poor reflectance of ecological knowledge needed to meet international and national policy goals, poor compliance with the certification standard, and limited use of sanctions in the case of poor compliance on the ground (SSNC 2010; J. Terstad and J. Rudberg pers. comm.). Also in other countries the poor representation of biodiversity conservation principles in forest certification schemes has been criticized (Bennett 2000; Ghazoul 2001). Thus, as stated by Tikina and Innes (2008), certification systems’ “effectiveness remains to be determined”. This suggests the need for assessments of the extent to which certification standards capture evidence-based knowledge and comply with agreed goals.

The application of FSC in Sweden forms an interesting case study regarding the extent to which evidence-based knowledge is utilized in the national FSC standard’s ecological indicators. Maintaining ecological sustainability has been a main driver for the transition from the sustained yield paradigm in forestry toward sustainable forest management (SFM) in Sweden (Bush 2010). This applies to the work with the Swedish FSC-standard, which began in 1993 after the Taiga Rescue Network conference in Jokkmokk 1992, and the establishment in Sweden of the first interim FSC standard in 1995 (Elliott and Schlaepfer 2001; Cashore et al. 2004). Additionally, the first FSC assessment of a forest management unit was made in Sweden in 1996 (Rhubes et al. 1996), and Sweden was the first country in the world to endorse a national FSC standard in 1998. FSC forest certification has a strong position in Sweden with more than 11 million hectares of forest land certified (see http://www.fsc-sverige.org). This is about half of all productive forest in Sweden and ~7 % of the area of FSC certified forests globally.

Due to a long history of effective sustained yield forestry (Angelstam et al. 2011a), Sweden is of particular interest for evaluating the impact of FSC on ecological sustainability. Being a latitudinally extended country with a diverse history of natural resource use, biodiversity has both natural forest and cultural woodland benchmarks (Angelstam 2006). While the areal extent of boreal forest in Sweden has been stable during the past century (Jansson 2011), the area of natural and near-natural forests has decreased considerably with the development of sustained yield wood production (Angelstam 1997). In the south, temperate and hemiboreal forests and woodlands have a long history of human management in the context of agroforestry, animal husbandry, and local use of wood and biomass. Here, scattered natural forest remnants and old trees in managed wooded grassland provide habitat for a large number of forest specialists. The introduction of sustained yield forestry based on planted Norway spruce (*Picea abies*) during the past century increased forest cover considerably (Jansson 2011). As a consequence of these transitions, declines in species’ distributions and abundances have been reported from a wide range of taxonomic groups (Gärdenfors 2010). The main driving mechanism is loss of natural habitats, whereby natural forest properties required by species have been reduced to inadequate amounts due to short rotation times and management to reduce the diversity and complexity of forests.

The general objective of this study is to assess to what extent FSC certification standards in Sweden can be expected to contribute to ecological sustainability. To achieve this purpose we compare ecological indicators in the Swedish negotiated certification standards with evidence-based knowledge about what is needed to measure and assess the state of ecological sustainability. First, a normative model is developed based on evidence-based knowledge about (a) composition, structure, and function at multiple scales underpinning the monitoring of ecosystems, and (b) performance targets derived by comparing the habitat amount in naturally dynamic forests, in managed forests, and that required for the persistence of species’ populations. Second, we compare the FSC certification standards’ ecological indicators from 1998 and 2010 in Sweden with evidence-based knowledge using the Specific, Measurable, Accurate, Realistic, and Timebound (SMART) indicator approach. Finally, we discuss limitations with negotiated certification standards to achieve ecological sustainability, and propose collaborative learning among stakeholders as an approach to realize the FSC vision of environmentally appropriate forest management.

## Methodology

### An Evidence-Based Normative Model

#### Measuring the State of Forest Ecosystems

Ecosystems can be described by their composition, structure, and function at different spatial scales (Noss 1990, see also Table S1 in Electronic Supplementary Material). Composition refers to the identity and variety of ecosystem components, including genetic diversity, species richness, and abundance, and the variety and amounts of biotopes in the landscape. Structure refers to the spatial arrangement of the various components of the ecosystem, such as the heights of different canopy levels and the spacing of trees in a stand or patches in a landscape. Function refers to various ecological processes, and the rates at which they occur. Therefore indicators describing ecosystems should represent the following aspects of ecosystems: (1) species and ecosystem components derived from species and biotopes (i.e. composition); (2) habitats as the spatial arrangement of various components found in naturally dynamic forests and pre-industrial woodland (i.e., structure); (3) processes such as primary production, decomposition, nitrogen cycling, hydrologic cycle, soil formation, natural disturbance, dispersal, and biological interactions among trophic levels (i.e., function) (e.g., Larsson et al. 2001; Angelstam and Dönz-Breuss 2004; Brumelis et al. 2011). In addition multiple spatial scales from tree and stand levels to landscapes and ecoregions need to be included.

#### Performance Targets as Norms for Assessment of Sustainability

Assessing ecological sustainability involves monitoring indicators and comparing their state with performance targets describing the states which are deemed sustainable. Focusing on the role of ecosystems as providers of natural capital, the naturalness concept is useful for defining benchmarks for sustainable ecosystems (Electronic Supplementary Material S2). For the conservation of species, non-linear responses of species to habitat loss can be used to formulate performance targets. To define how much habitat is enough for the persistence of species in the long-term, we made a review of knowledge with a focus on specialized focal species requiring old forest, downed dead wood, and standing dead wood (Electronic Supplementary Material S2, S3). The results show that available knowledge can be used to formulate evidence-based norms that define how much of forest properties are enough for species populations (Electronic Supplementary Material S3). They also point at large differences between, for example, on the one hand, the amounts of downed dead wood found in naturally dynamic forest ecosystems and the requirements of specialized focal species and, on the other, the amounts found in most of today’s managed forests (Fig. S2; Table S2).

### Analyses of Indicators Used in Swedish FSC Standards

Assessment consists of comparing parameter values of different indicators with norms, verifiers, or targets (Lammerts van Bueren and Blom 1997; Busch and Trexler 2003; Wismar et al. 2008). Our normative model states that compositional, structural, and functional indicators at multiple spatial scales are needed to measure ecological sustainability, and that there is empirical knowledge about how much habitat species need that could be used in FSC standards. We used the SMART^2^ approach (Wismar et al. 2008) to analyze the direct and indirect indicators related to ecological sustainability in the Swedish FSC standards from 1998 (*n* = 31) and 2010 (*n* = 81). We assessed whether or not the indicators were: (1) Specific, that is related to variables that monitor the status of compositional, structural, and functional ecosystem properties at one or more of three terrestrial spatial scales: trees in stands, stands in landscapes, and landscapes in ecoregions (Elbakidze et al. 2011), as well as aquatic and riparian; (2) Measurable, that is including clearly defined units (e.g., ha, m^3^/ha, %). Cashore (1997) differentiated between procedural and substantive policy types. We excluded indicators which cannot be used to measure ecological status but instead could be seen as procedural (such as setting aside sites with red-listed species) or that are linked to the governance of conservation or FSC certification (such as the implementation of procedures that promote a certain kind of management); (3) Accurate, that is with a target value or range of values; (4) Realistic, i.e., achievable as a short-term target towards a long-term goal representing evidence-based knowledge; (5) Timebound, that is with a statement about when the target should be reached. Whenever an indicator could be misunderstood, quantities (a number) or qualities (such as “all”, “long term”, or “never”) were not mentioned, or the terminology was vague, we did not include the indicator in the analysis, or gave it a lower score. We also evaluated the extent to which the indicator covered terrestrial and aquatic ecosystem’s composition, structure, and function at the scales of aquatic, riparian zone, trees in stands, stands in landscapes, and landscapes in ecoregion.

## Results

From the pool of ecologically relevant direct and indirect indicators in the 1998 standard we found 19 indicators (Table 1), and in the 2010 standard we found 23 that were useable for the analyses (Table 2). Terrestrial indicators dominated, except for one indicator relevant for riparian zones. We found no aquatic indicator. The number of terrestrial indicators that were specific enough to be attributed to composition, structure, and composition at any of the three terrestrial spatial scales increased from 1998 to 2010 (Tables 1, 2). More importantly, their identity related to several spatial scales and ecosystem dimensions increased. We could not identify any distinct landscape level indicator in the 1998 standard, but in 2010 two dealt with this scale. Similarly, one indicator explicitly dealt with ecosystem functions (fire) in the 1998 standard, but in the 2010 standard we found three, with two additional indicators linked to protective functions. While only one indicator fulfilled all five criteria of the SMART framework in 1998 and 2010, respectively, the number of indicators with higher levels of SMARTness increased (Table 3).

**Table 1:** State indicators in the Swedish FSC standard from 1998 that capture properties in terrestrial and riparian/aquatic forest ecosystems. An assessment of the SMARTness of each indicator is presented (see “Methodology” section). The interpretation of different part of the SMART criteria is shown in brackets (*S* specific, *M* measurable, *A* accurate, *R* realistic, *T* timebound)

Landscapes in ecoregion	Composition	Structure	Function
	NA	NA	NA
Stands in landscapes	4.2.3 arboreal lichens (S) 5.1 areas of virgin-type forests; exempt from forestry (S) 6.1.1a “un-even-aged and stratified forest”, quantitative target (“exempt”) (S) 6.1.1b Woodland Key Habitat, quantitative target (“exempt”) (S) 6.1.1c non-productive <1 m^3^ ha^−1^ year^−1^, quantitative target (“exempt”) (S) 6.1.2 exempt >5 % of productive forest area (SMA)	6.7.2 balanced age distribution for the landscape ecology, especially old forest if uncommon (S) 6.7.3 >5 % broad-leaved trees on mesic and moist sites (SMA)	6.4.4 Proportion of burned clear-felled areas, 5 % during 5 years (SMART)
Trees in stand	4.2.3 arboreal lichens (S) 5.2 strips and enclaves (S) 6.5.4 small habitats, patches, tree groups, special values (SM) 6.5.5 trees with biodiversity value (S) 6.5.6 Number of potential old and large trees, 10 per hectare (SMA) 6.5.7 fresh dead wood <3 m^3^ (SMA) 6.5.8 create standing dead wood (S)	6.5.12 broad-leaved trees during cleaning and thinning >5–20 % according to soil condition (SMA)	NA
Riparian	4.2.3 arboreal lichens (S) 6.5.4 small habitats, patches, tree groups, special values (SM)	NA	NA
Aquatic	NA	NA	NA

**Table 2:** State indicators in the Swedish FSC standard from 2010 that capture properties in terrestrial and riparian/aquatic forest ecosystems. An assessment of the SMARTness of each indicator is presented (see “Methodology” section). The interpretation of different part of the SMART criteria is shown in brackets (*S* specific, *M* measurable, *A* accurate, *R* realistic, *T* timebound)

	Composition	Structure	Function
Landscapes in ecoregion	6.4.2 consider landscape representativeness of 6.4.1 (S) 9.1.1a high conservation value forest (HCVF) concentrations (S(M))	NA	NA
Stands in landscapes	6.2.1a “un-even-aged and stratified forest”, quantitative target (“exempt”) (S) 6.2.1b Woodland Key Habitat, quantitative target (“exempt”) (S) 6.2.1b non-productive <1 m^3^ ha^−1^ year^−1^, quantitative target (“exempt”) (S) 6.2.5 document nests and capercaillie leks, and protect them (SM) 6.4.1 productive forest set-aside, proportion of landscape, 5 % (SMA) 9.1.1b sub-alpine HCVF (SM)	6.1.3 “balanced age distribution”, no quantitative target (S) 6.3.9 deciduous trees on mesic and moist sites, proportion of landscape, 5 % (SMA) 6.3.10 proportion of spruce-dominated stands, proportion of landscape, <50 % (SMAR) 6.3.19 promote broad-leaf and biodiversity value trees (S)	6.3.12 burn dry or mesic sites, proportion of regeneration area in the landscape during 5 years, >5 % (SMART) 9.1.1c protective forest (HCVF; §15 Forestry Act) (SM) 9.1.1d source of water supply (HCVF) (SM)
Trees in stand	3.2.2 arboreal lichens (S) 6.3.7 high stump or girdled trees, n ha^−1^, 3 of all tree species (SMAR) 6.3.14a demarcate small habitats (SM) 6.3.14b demarcate buffer zone (SM) 6.3.15 demarcations of transitions to wetlands and low productive sites, no unit, no target (SM) 6.3.16 wind resistant trees, n ha^−1^, 10 (SMAR)	6.3.8 broad-leaved trees, proportion of stand volume, 10 % and 5 % north of Limes Norrlandicus (SMAR)	NA
Riparian	3.2.2 arboreal lichens (S)	NA	NA
Aquatic	NA	NA	NA

**Table 3:** Number of FSC standard state indicators in Sweden and the extent to which they satisfy the SMART criteria. The numbers within brackets denote indicators that are close to fulfilling the criteria

Swedish FSC standard	Specific	+Measurable	+Accurate	+Realistic	+Timebound
1998					
Terrestrial	19	1 (7)	1 (5)	1 (5)	1
Riparian and aquatic	2	(1)	0	0	0
2010					
Terrestrial	23	5 (10)	5 (2)	5 (2)	1
Riparian and aquatic	1	0	0	0	0

Aquatic ecosystems were poorly represented. Nevertheless, in the FSC standard it is stated that forest managers shall implement procedures that promote continuously forested, if possible stratified, transition zones conditioned by topographical, hydrological and ecological features along watercourses and open water areas (criteria 6.5.14 in FSC standard 2010). Additionally, managers shall consider wetland and aquatic habitats in a watershed perspective beyond the context of the landholding and implement specific consideration measures in such habitats with high biodiversity values (criteria 6.5.17 in FSC standard 2010). A potential problem here is that these indicators are not using any clear definitions or numbers and thus can be interpreted in very different ways.

We could only identify four reasonably unambiguous negotiated performance targets. These were the area proportion of burned final felling areas (5 %) in the 1998 and 2010 standards, the number of girdled trees and high stumps (3 ha^−1^), stand volume proportion of deciduous trees (5–10 %), and the proportion of spruce-dominated stands (<50 %) in landscapes south of the natural distribution range of Norway spruce.

## Discussion

### Assessing Ecological Sustainability is Possible

Northern forest ecosystems are globally important for the maintenance of ecosystem services, for example, by providing wood, fiber, bioenergy, species, habitat, carbon sequestration, water cycling as well as cultural and recreational values (Burton et al. 2003; Gauthier et al. 2009; Parrotta and Trosper 2012). Measuring ecosystem properties is a humbling undertaking. However, over the past two to three decades, the pool of knowledge about composition, structure, and function of Fennoscandian forests has grown immensely as several reviews, research programs, and conferences have focused on forest biodiversity (e.g., Korpilahti and Kuuluvainen 2002; Angelstam et al. 2004; Villard and Jonsson 2009; Jonsson et al. 2011a). It should, however, be noted that there are fewer ecological benchmark data for hemiboreal than boreal forests. On the other hand, the forest companies that employ FSC certification operate mainly in the boreal biome in Sweden (Keskitalo et al. 2009).

A long history of forest management focusing on high and sustained yield (Eriksson et al. 2007) shifts the quantities of the compositional, structural, and functional elements of forest ecosystems at different spatial scales (Electronic Supplementary Material S2, S3). The amounts of terrestrial natural forest legacies such as dead wood, large trees, and old forest are one to three orders of magnitude lower in landscapes with a long forest history, than in naturally dynamic forests (Electronic Supplementary Material S2, S3). The length and intensity of forest use thus affect the degree of deviation from a forest regions’ natural range of variability (NRV) (Angelstam et al. 2013). Because in parts of Sweden silviculture for sustained yield wood production began almost two hundred years ago (Angelstam et al. 2011a), the deviation from NRV is much larger than, for example, in many boreal forest regions in Russia (Shorohova and Tetioukhin 2004) and Canada (Cyr et al. 2009). Results from forest modeling (Pennanen 2002), forest history (Angelstam et al. 2013), and comparative studies of forest landscapes (Roberge et al. 2008; Müller and Bütler 2010) present a similar pattern, which indicates that this conclusion is robust. Regarding riparian and aquatic ecosystems there is limited evidence-based knowledge, both with respect to NRV and managed forest range of environmental variables, but even less with regard to threshold values for specialized, endangered or focal species in riparian and aquatic environments.

The large difference between managed and natural landscapes suggest that to satisfy current policies about biodiversity conservation and ecosystem services, there is a need for the restoration of compositional, structural, and functional ecosystem components in managed terrestrial and aquatic ecosystems (e.g., Burton and Macdonald 2011). Improving the matrix around protected areas by retention forestry (Gustafsson et al. 2012), management of ecosystem engineers (Törnblom et al. 2011), and regulation of herbivore densities (Hothorn and Müller 2010) are good examples.

The occurrence and fitness of species and functionality of various processes may exhibit step functions or thresholds in their response to habitat, which has obvious implications for the formulation of conservation targets (Andersen et al. 2008; Villard and Jonsson 2009). This is the same as dose–response curves or thresholds of disease eradication used in health sciences (Anderson and May 1991). Evidence-based knowledge about the requirements of forest-reliant species is indeed accumulating, and some of that knowledge has been validated by comparing predictions based on empirical studies with independent data (Angelstam et al. 2004; Edman et al. 2011). Even if there is very large variation in the amount of habitat required by species, it is clearly higher than what is present in most managed landscapes (Electronic Supplementary Material S3, Fig. S2). For example, the amount of dead wood required on average across species or communities is similar (20–40 m^3^ ha^−1^) in the three major forest biomes from lowland nemoral broadleaf forests, mountain areas in continental Europe and boreal forests (Müller and Bütler 2010). Interestingly, the range of common thresholds values from northern Europe is similar to other ecosystems. For example, in South America, Mordecai et al. (2009) showed that both habitat occupancy and use showed strong threshold responses at 21–40 % upper canopy cover. Nevertheless, the level of knowledge for deriving ecological targets is still incomplete for many species groups, forest ecoregions, disturbance types, and successional stages. Moreover, knowledge is limited about the link between genetic diversity and ecosystem functions in areas and regions with different histories of land use (Bihn et al. 2010).

### FSC Indicators Are Not SMART and Negotiated Targets Few

To our knowledge there is only one study that compares FSC indicators with ecological knowledge in Sweden. Focusing on the 1998 FSC standard and its use of ecological knowledge, Dahl (2000) concluded that “Although the FSC-standard is the first step towards environmentally appropriate forestry, there is still a long way to go before the biodiversity of the forests is secured…”. The Swedish FSC standards from 1998 and 2010 contained indicators concerning compositional, structural, and functional ecosystem properties at multiple scales, but very few included unambiguous performance targets. Only one of 19 indicators in 1998 and one of 23 indicators in 2010 satisfied all five SMART criteria. Very few indicators were related to riparian and aquatic ecosystems.

Nevertheless, the Swedish FSC standard process has evidently resulted in some learning and subsequent inclusion of a wider coverage of spatial scales, i.e., moving from trees and stands to also include landscapes in ecoregions. In addition we observed a slight expansion of the thematic cover from 1998 to 2010. There are, however, mismatches between the indicators and what needs to be covered to measure ecosystems in terms of structure and function at the scale of landscapes in regions (see also Elbakidze et al. 2011). This applies in particular to aquatic systems.

This study thus shows that even if there are gaps regarding evidence-based knowledge of how to define ecological sustainability, the mismatch between existing evidence-based knowledge and what is applied in the Swedish FSC standard is large. This is not surprising as FSC has the character of a social process, and not an evidence-based collaborative learning process with the aim to reach all dimensions of SFM. In particular, ecological sustainability is only one of several criteria to be considered in standard negotiation processes. Nevertheless, ecological indicators are usually the primary ones that have a chance of adhering to SMART criteria. By contrast, the social and economic and legal requirements are often much more value-based (i.e., not evidence-based), and they tend to be more difficult to measure with any precision. They are often more about relationships (public consultation, stakeholder rights, etc.) and subjective assessments of these things are often the norm in audits.

Also other reviews of negotiated and evidence-based conservation targets have observed clear differences between these two approaches to formulation of assessment norms. Based on a review of 159 articles reporting or proposing 222 conservation targets, Svancara et al. (2005) assessed differences between policy-driven and evidence-based approaches for defining the area percentages to be allocated for conservation. On average the proportion of area recommended based on evidence-based studies in terms of conservation assessments (31 %) and threshold analyses (42 %) were almost three times as high as those recommended as a result of negotiation processes (13 %). Similarly, the Natura 2000 process became in Germany a negotiated policy-formulation process with a mismatch with current evidence-based conservation targets. While the optimal habitat condition in managed beech forests was set at 10 m^3^ ha^−1^ dead wood, evidence-based studies suggest that 30 m^3^ ha^−1^ of dead wood is needed (Winter and Seif 2011). The presence of thresholds has also been a key concern in other standard setting processes, such as that of the FSC in British Columbia (Cashore et al. 2004).

Currently European forests are variable in their conservation value, with high values in those areas with a concentration of natural legacies linked to a shorter use history, and low values where forest use has been long and intensive. Regarding the FSC standard, do required performance targets apply to every hectare of forest, or should there be a concentration of efforts to some specific areas? Because habitat size and connectivity are two key aspects of species conservation, the functionality of habitat networks needs to be assessed at different scales from tree and stand to landscape and regional levels (e.g., Elbakidze et al. 2011). One approach would be to define different performance targets for different parts of landscapes and regions, instead of spreading a too thin layer of conservation efforts evenly and everywhere. This is consistent with the TRIAD approach comprised of extensive and intensive forestry and protected areas in different zones (MacLean et al. 2009). However, in Sweden the current system of forest ownership and governance largely precludes the implementation of such an active spatial planning approach (Eriksson and Hammer 2006; Angelstam et al. 2011b).

FSC is a widely applied certification brand in the boreal biome (Keskitalo et al. 2009; Elbakidze et al. 2011), which certifies that forest products have been produced in a responsible way in line with a higher environmental ambition level than policies and laws in the respective country (Pattberg 2005). In the long run, we argue that successful implementation of this marketing tool for “green” products (Kärnä et al. 2003) requires that FSC certification sends a consistent message to both stakeholders and customers about the extent to which certification contributes to forests’ ecological sustainability. Ultimately, this calls for harmonization of national standards’ indicators among countries and regions with similar ecosystems so that they better mirror evidence-based ecological knowledge that maintains ecological sustainability with an agreed ambition level, and produce desired results on the ground.

### Can Evidence-Based Knowledge be Included in Standards?

The mechanism for FSC standard revision is regulated in a standard procedure (FSC 2009a), and a FSC standard should be reviewed every 5 years. Revising the first Swedish certification standard from 1998 took 12 years. The revision process of the Swedish 2010 standard began in March 2012. However, the limited emphasis on evidence-based knowledge in negotiated standards stresses the need for systematic evaluation of the process of implementing policies about ecological sustainability, and learning to allow for gradual revisions that better mirror evidence-based knowledge (cf. Svancara et al. 2005; Tear et al. 2005).

Policy-driven norms represent the net results of different stakeholders’ views and agendas. Thus, the outcome of formulations and revisions of any norm, such as FSC standards, are likely to mirror national and regional differences in coupled human and nature systems as well as their history. Examples include, but are not limited to, forest history (Angelstam et al. 2011a), forest industrial regimes, and the related power relationships among stakeholders and societal choice (Lehtinen et al. 2004). This means that there are a multitude of factors other than evidence-based knowledge that affect standard negotiation outcomes. These factors are captured by Max Weber’s typology of social action, which includes four main types: (1) Rational action: action with a purpose to achieve a (given) goal (outcome). Examples include economy, government, technology and in general how human individuals make use of expectations as a means to reach their preferred ends. (2) Value-based action: Value oriented action, involving a belief in the absolute value such as ethical, esthetic, and religious values over the prospects of a successful result of the action itself. (3) Emotional actions: Actions based on the emotions determined by the affects and feelings of the person. (4) Traditional actions: Actions based on customs and practice (Weber 1922; Parsons 1949). As noted by Gulbrandsen (2008) scientific information usually has little influence when strong economic counter-forces are involved in the decision-making process. This means that even if evidence-based ecological knowledge might be introduced as a part of the negotiation process leading to a standard, there is no guarantee that it will be used. However, this problem may be ameliorated by facilitating co-production of knowledge among scientific experts, practitioners, and decision-makers.

Finally, it should be noted that voluntary forest certification is not the only tool used in Sweden with the aim to contribute to the ecological sustainability of forests and woodlands. Additionally, the selection of silvicultural systems in relation to site conditions and ecoregion (Puettmann et al. 2009), the implementation of retention forestry (Gustafsson et al. 2012), the development of formally protected area networks (Angelstam et al. 2011b), the contribution from voluntary protection by non-certified forest owners, the development of landscape planning approaches (e.g., Fries et al. 1998) and the level of collaboration with the aim to secure functional habitat networks in the landscape among different actors and stakeholders are important. However, this requires that forest land owners and managers, the state, as well as other stakeholders understand the effectiveness of and contribution from each tool, and plan accordingly with the aim to develop and maintain the level of forest composition, structure and function required to maintain ecological sustainability at multiple spatial scales from trees in stands to landscapes in ecoregions.

Currently, focus areas in European forestry include energy, economy, and safety (Anon. 2011). Thus “…imminent challenges facing the forest sector in Sweden and other European countries is to meet the anticipated increasing demand for wood raw materials resulting from the promotion of renewable energy sources” (Jonsson et al. 2011b). In addition there are stakeholders representing a multitude of forest owner categories and other interests such as biodiversity conservation, cultural heritage preservation, intensive forest management, forest industry, rural development, hunting, labor rights, and indigenous people’s rights. The meaning of the term forest sector is thus broadening considerably (Beland Lindahl and Westholm 2011). Hence, there is a need to measure and assess the aggregated effects of certification and other tools aiming at development toward sustainability of forests at multiple scales (e.g., Elbakidze et al. 2011), develop decision-support systems (Sandström et al. 2011), and for an informed collaboration (e.g., Axelsson et al. 2011). However, the FSC standard is limited to the landowner as the certificate holder. In Sweden, forest owner categories differ with respect to their conservation policy ambitions (Andersson et al. 2012). Thus, areas with many land owners or land ownership categories represent a major challenge to achieving ecological sustainability across landscapes and ecoregions (Sandström et al. 2011).

## Electronic supplementary material

Below is the link to the electronic supplementary material.

Supplementary material 1 (PDF 134 kb)

## Footnotes

1
http://www.fsc.org.

2 The SMART approach used in thus study is different from the one used by FSC itself (FSC 2009b).
