# Supplementary material for: Evidence-Based Knowledge Versus Negotiated Indicators for Assessment of Ecological Sustainability: The Swedish Forest Stewardship Council Standard as a Case Study
Source: Ambio. 2013 Mar 10;42(2):229–40. doi: 10.1007/s13280-012-0377-z (PMC3593031; doi:10.1007/s13280-012-0377-z)
Supplement: Supplementary file 1 — Supplementary material 1 (PDF 134 kb) [file 13280_2012_377_MOESM1_ESM.pdf]

Electronic Supplementary Material

Evidence-based versus negotiated knowledge for assessment of ecological sustainability: the Swedish Forest Stewardship Council standard as a case study

Per Angelstam, Jean-Michel Roberge, Robert Axelsson, Marine Elbakidze, Karl-Olof Bergman, Anders Dahlberg, Erik Degerman, Sönke Eggers, Per-Anders Esseen, Joakim Hjältén, Therese Johansson, Jörg Müller, Heidi Paltto, Ihor Soloviy, Tord Snäll, Johan Törnblom

S1. FSC in the context of sustainable forest management

According to the UN (2008) sustainable forest management (SFM) as a dynamic and evolving concept aims to maintain and enhance the economic, social and environmental value of all types of forests, for the benefit of present and future generations. It is characterized by seven elements (i.e. criteria), including: (1) extent of forest resources; (2) forest biological diversity; (3) forest health and vitality; (4) productive functions of forest resources; (5) protective functions of forest resources; (6) socio-economic functions of forests; and (7) legal, policy and institutional framework (UN 2008). This vision is clearly pronounced in forest-related policy at multiple levels (e.g., MCPFE 2003; Forest Europe 2011). Policy implementation instruments to maintain, restore or improve ecological sustainability include (1) market-based incentives such as certification, (2) command and control measures such as regulation of silvicultural practices, and (3) government production or expenditure as well as state subsidies and taxes (e.g., Eskeland and Jimenez 1992; Sterner 2003).

Forest certification involves several stakeholder groups including forest owners and managers, non-governmental organizations, producers of forest products, consumers, and the certification body itself. Because of growing environmentally sensitive markets, businesses use forest certification as a market tool to become accepted by ecologically aware customers providing higher (so called "green premium") or more stable prices (e.g., Kärnä et al. 2003). Being a market tool, forest certification aims at providing support and better access to markets, especially environmentally sensitive ones. Significant attention is paid to ecological footprint related to the origin of forest products; to protect access of producers to resources, to decrease negative ecological, economic and social impacts and as such the risks coming from forest management interventions into the forest ecosystem. Introduction of forest certification aims to increase consumers' trust of certified products and formation of a favorable investment climate in the forest sector (Soloviy et al. 2009). Certification standards attempt to regulate what actions should be taken and what should be achieved, and the standard negotiation process favors mutual learning and understanding among multiple stakeholders and finally the acceptance of the negotiated, consent-based standards.

Thus, FSC can be viewed as a tool that brings the opportunity to contribute to implementation of ecological sustainability by considering evidence-based knowledge about ecosystems in the context of SFM policy. Developed indicators that are required by the biodiversity policy implementation process should be seen as describing a policy implementation feedback loop that begins with a pressure (i.e. resource consumption, overexploitation and climate change impacts) leading to a state (i.e., extinction risk, habitat extent and condition, and community composition) and resulting in a response (i.e., coverage of protected areas, sustainable forest management, policy responses) (see Butchart et al. 2010). This study focuses on state indicators for forest ecosystems because only such indicators can be used to measure the actual level of ecological sustainability.

References

Butchart, S.M.H., M. Walpole, B. Collen, A. van Strien, J.P.W. Scharlemann, R.E.A. Almond, J.E.M. Baillie, B. Bomhard et al. 2010. Global biodiversity: Indicators of recent declines. *Science* 328: 1164-1168.

Eskeland, G.S., and E. Jimenez. 1992. Policy instruments for pollution control in developing contries. *The World Bank Research Observer* 7(2): 145-169Forest Europe 2011

Kärnä, J., E. Hansen, and H. Juslin. 2003. Environmental activity and forest certification in marketing of forest products – a case study in Europe. *Silva Fennica* 37(2): 253–267.

MCPFE. 2003. *Improved Pan-European indicators for sustainable forest management*. Ministerial Conference on the Protection of Forests in Europe. Vienna: Liaison Unit.
Soloviy, I., V. Kovalyshyn, and F. Cubbage. 2009. Forest Certification in Europe and North America. Building Insights of Managerial Economics and Accounting Toward Sustainable Forest Management. Proceedings of IUFRO Conference, Lviv, Ukraine: Ukrainian National Forestry University.
Sterner, T. 2003. *Policy instruments for environmental and natural resource management*. Washington DC: Resources for the Future.
UN. 2008. Resolution 62/98, establishing the Non-Legally binding instrument on all types of forests. Retrieved 2012-11-22 at http://www.un.org/ga/search/view_doc.asp?symbol=A/RES/62/98&Lang=E

S2. Development of normative model

Monitoring

Composition, structure and function of ecosystems form the foundation for measuring ecological sustainability (see Table S1). Important natural forest components include dead wood (Siitonen 2001), deciduous trees, old trees, understorey and stands of naturally dynamic forest (e.g., Berg et al. 1994; Rouvinen et al. 2002; Nilsson and Wardle, 2005; Fedrowitz et al. 2012). Habitat specialists are especially vulnerable to reduced amounts of these components (e.g., Schmiegelow et al. 1997; Owens and Bennett 2000). For instance, altered structure such as different size and decay stage of dead wood (Stokland 2001), loss of multi-layered forests (Eggers et al. 2005), edge creation by forestry (Jansson et al. 2011) and fragmentation of natural forest habitats (Edman et al. 2011) all affect species' demography, abundance and diversity. Additionally, functions including natural disturbances such as fire (Zackrisson 1977) and browsing (Angelstam et al. 2000) have been altered over a long time.

Table S 1 Description of the terms composition, structure and function in the context of ecological sustainability (from Larsson et al. 2001; see Angelstam and Dönz-Bruess 2004 for examples of measurable variables).

	Description
Composition	The identity and variability of elements in a collection, and includes lists and measures of ***species*** and ***genetic diversity***. Ecosystems are composed of organisms, species, groups of closely interacting species, genetic diversity within species, legacies of species (e.g., dead wood and soil organic matter), and various inorganic components (e.g., minerals and gases)
Structure	The physical organization or ***pattern*** of a system, from habitat complexity as measured within communities, to the pattern of patches and other elements in landscapes. Ecosystem structure arises from the patterns in which components of composition occur and includes architectural and social aspects. Architecture denotes the physical aspects of structure, such as the number of canopy layers, or patchiness of species and age classes. Social structure refers to patterns in the way that individuals, species or groups of species relate to each other, and to the system as a whole, such as predation, symbiontic relationships or mutualism.
Function	Ecological and evolutionary ***processes***, including gene flow, disturbances and nutrient cycling. There are two aspects of function: (a) the influence on processes (e.g., photosynthesis, nutrient cycling, population growth), and (b) the influence on ecosystem structure (e.g., balance among different populations). In addition to the internal functions of ecosystems, there are external functions, which are influences of the community as a whole on its surroundings. Examples include regulation of water and nutrient fluxes, stabilization of soils, and absorption and reflection of solar energy (albedo).

Naturalness: a key concept to describe reference ecosystems
The ecosystem concept was formally coined in the 1930s (Tansley 1935), although insights about ecosystems and their role as natural capital for society are widespread in traditional knowledge (Parrotta and Trosper 2012). An ecosystem consists of a biological community, and the physical and chemical factors that make up its non-living or abiotic environment. Policies about maintenance of forests, biodiversity and ecosystem services make explicit reference to the naturalness concept (MCPFE 2003). However, many current definitions of forest and natural that are applied in the context of forest statistics overlook ecologically important components of natural forests such as dead wood, old and large trees (Rouvinen and Kouki 2008). In spite of the ambiguity of this concept (e.g., Balée 1998; Egan and Howell 2001), indicator variables addressing ecological sustainability should represent both naturally dynamic forest ecosystems (Peterken 1996; Angelstam and Dönz-Breuss 2004; Brumelis et al. 2011) and pre-industrial agroforestry systems (Angelstam 2006). Other terms used to describe the conditions in benchmark or reference areas are ecological integrity (Pimentel et al. 2000), natural range of variation (NRV; Cyr et al. 2009), and historic range of variation (HRV; Egan and Howell 2001). Because the focus of this study is on natural forests and not cultural woodlands, in the following the focus in this study is on NRV and not HRV.

Reviewing evidence-based knowledge about species
Habitat loss is the main reason for species extinctions (e.g., Wilcove et al. 1998) and for the alteration of processes that impair delivery of ecosystem services (MEA 2005; Kumar 2010). The existence of non-linear responses of species and processes to habitat loss (e.g., Fahrig 2001, 2002) allow for the design of habitat-species studies to formulate evidence-based targets regarding how much of ecosystem attributes can be lost without losing species and populations, or altering natural processes (e.g., Muradian 2001; Angelstam et al. 2004a; Huggett 2005; Lindenmayer and Luck 2005; Villard and Jonsson 2009; Müller and Bütler 2010). This requires a systematic analytic approach to collecting empirical data using multiple methods (Angelstam et al. 2004b; Roberge and Angelstam 2009).

First, the range of variation of parameter values for habitat variables need to be identified by comparing naturally dynamic forests and managed forests with different management histories and thus different amount of suitable habitats. Then hypotheses about non-linear responses of species to habitat amounts can be tested through empirical studies comparing habitat amounts to the occurrence or viability of species populations (e.g., Angelstam et al. 2004a; Roberge et al. 2008). Similarly, historical data about habitat variables and species can be analyzed (Egan and Howell 2001).

Naturally dynamic forest ecosystems have shaped species and populations by natural selection and therefore provide an opportunity to learn about the NRV (e.g., Nilsson et al. 2007). Consequently, the benchmark for conservation of ecological sustainability at multiple spatial scales needs to be understood for different disturbance regimes (e.g., Shorohova et al. 2009; 2011; Kuuluvainen and Aakala 2011; see Fig. S1a), and their natural range of parameter values needs to be identified (Angelstam et al. 2004a; Roberge and Angelstam 2009; see Fig. S1b). Parameter values corresponding to NRV may directly provide conservation targets for very large, unmanaged areas where natural processes are allowed to occur (Cyr et al. 2009; Gauthier et al. 2009). However, in most parts of Europe forests are managed to produce raw material for industrial use, and thus depart considerably from NRV (e.g., Kouki et al. 2001; Angelstam and Dönz-Breuss 2004; Kuuluvainen 2009; see Fig. S1c). Hence, under such conditions NRV may not be a desired aim as it would not satisfy social and economic dimensions of sustainable forest management policy (e.g., Forest Europe 2011). By

contrast, in Quebec, Canada, the forest policy vision is to ensure that the age structure and spatial patterns of natural forests is preserved (Ministère des Ressources naturelles et de la Faune 2010). An alternative approach, such as the Swedish forest and environmental policy, is to address the quantitative requirements of naturally occurring specialized species as key ecosystem variables (see Bush 2010; Angelstam et al. 2011). Policies may thus represent different levels of ambition for biodiversity conservation, which each can be matched with evidence-based knowledge. This study focuses on a low level of ambition, presence of species. Other progressively higher levels include population viability, ecological integrity and resilience.

Admittedly, quantitative ecological knowledge about habitat requirements is insufficient for most forest species (e.g., Müller and Bütler 2010). Furthermore, the range of requirements is strongly dependent upon the traits of species, ranging from for example flightless saproxylic beetles to strong flying pyrophilic species and large mammals. However, emerging knowledge about the requirements of specialized (Angelstam et al. 2004a), ecologically important (Drapeau et al. 2009) or so-called focal or umbrella species (Roberge and Angelstam 2004) provide a useful starting point for formulating evidence-based performance targets for ecological sustainability (Fig. S1d). Due to the inherent uncertainties in empirical knowledge, these targets will generally take the form of ranges (i.e. intervals) rather than precise values. Finally, negotiated targets need to be compiled (Fig. S1e), and compared with evidence-based performance targets representing either NRV (Fig. S1b) or focal species' requirements (Fig. S1d). We emphasize that this knowledge should ideally be based on studies that link habitat structure and vegetation management to demographic parameters because habitat use (presence/absence or densities) may not reflect habitat quality (e.g., Van Horne 1983).

To review this complex knowledge, scientists involved with research about composition, structure and function of terrestrial and aquatic ecosystems in European forests were gathered. The group included experts of different taxonomic groups of species, habitats and ecosystem processes in terrestrial and aquatic ecosystems, in both managed and naturally dynamic forests. To extract evidence-based knowledge about how much habitat is enough we reviewed the literature for original work, reviews and meta-analyses of published literature. To make quantitative data about different forest ecosystem variables comparable, data for each variable (such as dead standing wood or forests older than final felling) was normalized by dividing all values with the highest observed value for that variable. Then the distribution of values in 5% units was used to illustrate differences between NRV, target intervals for species persistence and the situation in forests with a long management history.

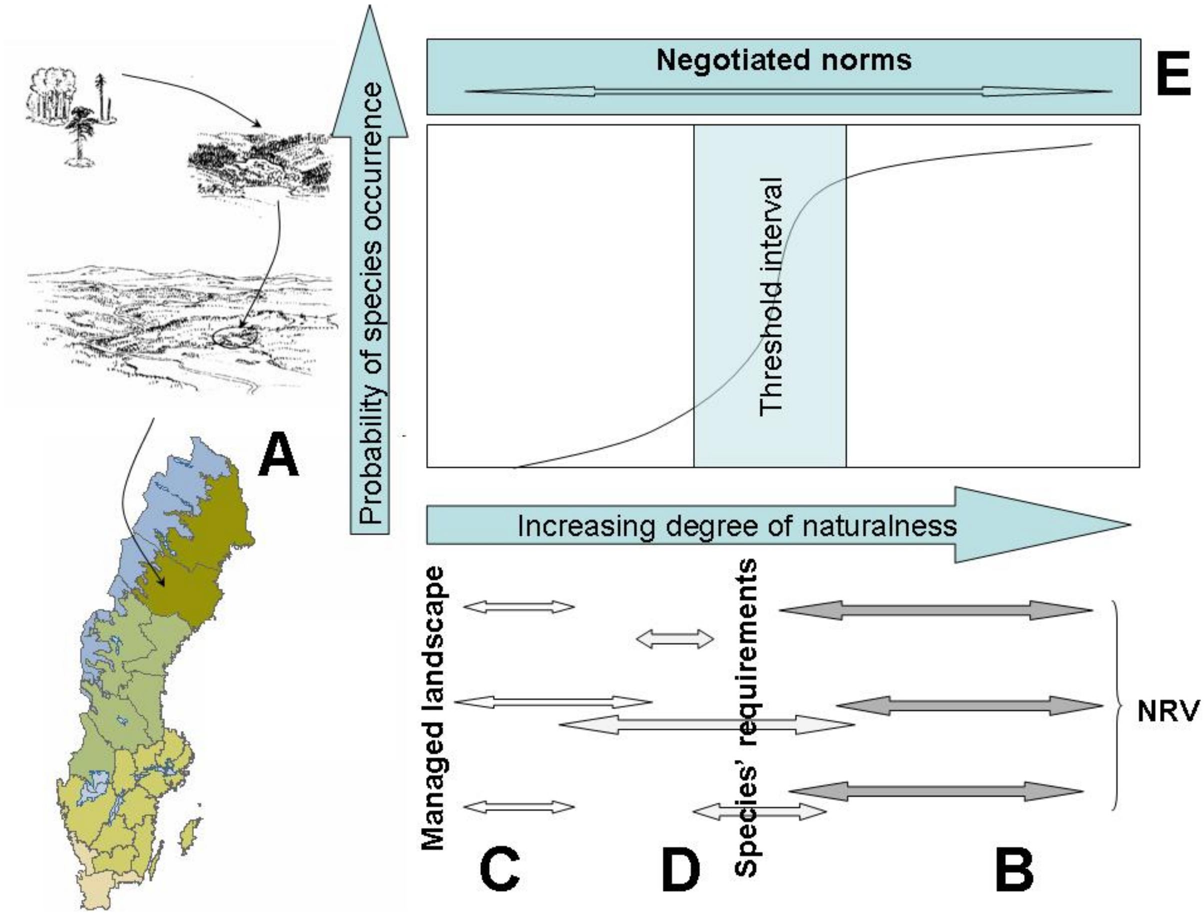

Fig. S1 Illustration of the methodology to derive performance targets for habitat amount. To describe ecosystems, several scales (trees in stands, stands in landscapes, landscapes in regions) need to be included (A). To understand the extent to which forest landscapes with different forest histories have sufficient amounts of habitat for the maintenance of species at different spatial scales, it is necessary to find out both what the natural range of variability is (NRV) (B), how much of this is left (C) and how much specialized species require (D). Finally, there is a range of negotiated policy-driven norms (E) that evidence-based targets can be compared with.

References

Angelstam, P. 2006. Maintaining cultural and natural biodiversity in Europe's economic centre and periphery. In *The Conservation of cultural landscapes,* ed. M. Agnoletti, 125-143. Wallingford: CAB International.

Angelstam, P., K. Andersson, R. Axelsson, M. Elbakidze, B.G. Jonsson, and J.M. Roberge. 2011. Protecting forest areas for biodiversity in Sweden 1991-2010: policy implementation process and outcomes on the ground. *Silva Fennica* 45(5): 1111–1133.

Angelstam, P., and M. Dönz-Breuss. 2004. Measuring forest biodiversity at the stand scale – an evaluation of indicators in European forest history gradients. *Ecological Bulletins* 51: 305-332.

Angelstam, P., M. Dönz-Breuss, and J.M. Roberge. 2004a. Targets and tools for the maintenance of forest biodiversity. *Ecological Bulletins* 51: 11-24.

Angelstam, P., S. Boutin, F. Schmiegelow, M.A. Villard, P. Drapeau, G. Host, J. Innes, G. Isachenko et al. 2004b. Targets for boreal forest biodiversity conservation – a rationale for macroecological research and adaptive management. *Ecological Bulletins* 51: 487-509.

Balée, W. 1998. *Advances in historical ecology*. New York, USA: Columbia University Press.
Brumelis, G., B.G. Jonsson, J. Kouki, T. Kuuluvainen, and E. Shorohova. 2011. Forest naturalness in northern Europe: perspectives on processes, structures and species diversity. *Silva Fennica* 45(5): 807-821.
Bush, T. 2010. Biodiversity and sectoral responsibility in the development of Swedish forestry policy, 1988-1993. *Scandinavian Journal of History* 35(4): 471-498.
Cyr, D., S. Gauthier, Y. Bergeron, and C. Carcaillet. 2009. Forest management is driving the eastern North American boreal forest outside its natural range of variability. *Frontiers in Ecology and Environment* 7(10): 519–524.
Drapeau, P., A. Nappi, L. Imbeau, and M. Saint-Germain. 2009. Standing deadwood for keystone bird species in the eastern boreal forest: Managing for snag dynamics. *Forestry Chronicle* 85:227-234.
Egan, D., and E.A. Howell. 2001. *The Historical Ecology Handbook: a Restorationists' Guide to Reference Ecosystems*. Washington. DC: Island Press.
Eggers, S., M Griesser, and J. Ekman 2005 Nest predation and habitat change interact to influence Siberina jay numbers. *Oikos* 111:150-158.
Fahrig, L. 2001. How much habitat is enough? *Biological Conservation* 100(1): 65-74.
Fahrig, L. 2002. Effect of habitat fragmentation on the extinction threshold: a synthesis. *Ecological Applications* 12(2): 346-353.
Forest Europe. 2011. *State of Europe's Forests*. Ministerial Conference on the Protection of Forests in Europe. Oslo: Liaison Unit.
Gauthier, S., M.A. Vailliancourt, A. Leduc, L. De Grandpré, D. Kneeshaw, H. Morin, P. Drapeau, and Y. Bergeron. 2009. *Ecosystem management in the boreal forest.* Quebec: Presses de l'Université du Quebec.
Huggett, A.J. 2005. The concept and utility of 'ecological thresholds' in biodiversity conservation. *Biological Conservation* 124(3): 301–310.
Kouki, J., S. Löfman, P. Martikainen, S. Rouvinen, and A. Uotila. 2001. Forest fragmentation in Fennoscandia: Linking habitat requirements of wood-associated threatened species to landscape and habitat changes. *Scandinavian Journal of Forest Research Supplement* 3: 27-27.
Kumar, P. 2010. *The economics of ecosystems and biodiversity. Ecological and economic foundations*. London and Washington DC: Earthscan.
Kuuluvainen, T. 2009. Forest management and biodiversity conservation based on natural ecosystem dynamics in northern Europe: the complexity challenge. *Ambio* 38(6): 309-315.
Kuuluvainen, T, and T. Aakala. 2011. Natural forest dynamics in boreal Fennoscandia: a review and classification. *Silva Fennica* 45(5): 823-841.
Lindenmayer, D.B., and G. Luck. 2005. Synthesis: Thresholds in conservation and management. *Biological Conservation* 124(3): 351–354.
MEA (Millenium Ecosystem Assessment). 2005. *Ecosystems and human well-being: Our human planet. Summary for decision makers.* Washington DC: Island Press.
MCPFE. 2003. *Improved Pan-European indicators for sustainable forest management*. Ministerial Conference on the Protection of Forests in Europe. Vienna: Liaison Unit.
Ministère des Ressources naturelles et de la Faune. 2010. Consultation on sustainable forest management in Québec. Gouvernement du Québec, 94 pp.
Müller, J., and R. Bütler. 2010. A review of habitat thresholds for dead wood: a baseline for management recommendations in European forests. *European Journal of Forest Research* 129: 981–992.
Muradian, R. 2001. Ecological thresholds: a survey. *Ecological Economics* 38: 7–24.

Nilsson, C., R. Jansson, B. Malmqvist, and R.J. Naiman. 2007. Restoring riverine landscapes: the challenge of identifying priorities, reference states, and techniques. *Ecology and Society 12(1): 16.*
Owens, I.P.F., and P.M. Bennett. 2000. Quantifying biodiversity: a phenotypic perspective. *Conservation Biology* 14(4): 1014-1022.
Parrotta, J., and R. Trosper. 2012. *Traditional forest-related knowledge: sustaining communities, ecosystems and biocultural diversity*. Dordrecht: Springer.
Peterken, G.F. 1996. *Natural Woodland – ecology and conservation in northern temperate regions.* Cambridge: Cambridge University Press.
Pimentel, D., L. Westra, and R.F. Noss. 2000. *Ecological integrity: Integrating environment, conservation, and health.* Washington DC: Island Press.
Roberge, J.M., and P. Angelstam. 2004. Usefulness of the umbrella species concept as a conservation tool. *Conservation Biology* 18(1): 76-85.
Roberge, J.M., and P. Angelstam. 2009. Selecting species to be used as tools in the development of forest conservation targets. In *Setting conservation targets for managed forest landscapes,* ed. M.A. Villard, and B.G. Jonsson, 426 pp. Cambridge: Cambridge University Press.
Roberge, J.M., P. Angelstam, and M.A. Villard. 2008. Specialised woodpeckers and naturalness in hemiboreal forests - deriving quantitative targets for conservation planning. *Biological Conservation* 141: 997-1012.
Rouvinen, S., and J. Kouki. 2008. The natural northern European boreal forests: unifying the concepts, terminologies, and their application. *Silva Fennica* 42(1): 135–146.
Shorohova, E., T. Kuuluvainen, A. Kangur, and K. Jõgiste. 2009. Natural stand structures, disturbance regimes and successional dynamics in the Eurasian boreal forests: a review with special reference to Russian studies. *Annals of Forest Science* 66(2): article 201.
Shorohova, E., D. Kneeshaw, T. Kuuluvainen, and S. Gauthier. 2011. Variability and dynamics of old-growth forests in the circumboreal zone: implications for conservation, restoration and management. *Silva Fennica* 45(5): 785-806.
Tansley, A.G. 1935. The use and abuse of vegetational terms and concept. *Ecology* 16(3): 284–307.
Van Horne, B. 1983. Density as a misleading indicator of habitat quality. *Journal of Wildlife Management* 47: 893–901.
Villard, M.A., and B.G. Jonsson. 2009. *Setting conservation targets for managed forest landscapes.* Cambridge: Cambridge University Press.
Wilcove, D.S., D. Rothstein, J. Dubow, A. Phillips, and E. Losos. 1998. Quantifying threats to imperiled species in the United States. *BioScience* 48: 607-615.

S3. How much habitat is enough?

The range of parameter values for ecosystem components under assumed NRV conditions, species requirements and amounts in managed forests showed large variation, but are still clearly different from each other (Table S3, Fig. S2). Variables capturing natural forest components varied the most under NRV, and the least in managed forests. The shape of the curve under NRV conditions suggests that about 30% of the reviewed studies of naturally dynamic landscapes have similar amounts of natural components as managed forests, and the other ones considerably more. However, about 75% of the studies in managed forests had <5% of the amounts under NRV conditions. Finally, of the reported threshold values or intervals for species populations, 80% were in the range of 10-30% of NRV condition. However, it is important to recognize that some species require amounts of natural components found only in the higher end of the range of variation.

Table S2 References reviewed regarding terrestrial ecosystem components (old forest, downed dead wood, standing dead wood) representing estimates about the variability in naturally dynamic forests, managed forests and evidence-based knowledge about the requirements of specialized focal species' needs (data sufficient only for downed dead wood)

Managed forest	Requirements of specialized species of conservation concern or species assemblages	Natural range of variability
3-5% old-growth forest (Angelstam and Andersson 2001:46)<br>7% of state forest in boreal Sweden >150 years in 1990 (excluding mountain forest) (Linder and Östlund 1998).<br>1-11% older forest stands (>120-150 years) in the same three study landscapes in 1990 (Linder and Östlund 1998).<br>0.1-2.1% old forest (Franc et al. 2007)<br>1-13% decidous forest in landscape (Mikusinski et al. 2003:523)<br>0-21% decidous forest in landscape (Bütler et al. 2004a)<br>0.5-9% decidous forest in landscape (Franc et al. 2007)<br>4.5 m^3ha^{-1} (CV=2.7%) downed dead wood in managed productive forest across Sweden (Fridman and Walheim 2000).<br>0.4-1.1 m^3ha^{-1} downed dead wood (Bütler et al. 2004a)<br>2.1-4.0 m^3ha^{-1} downed dead wood (Angelstam and Dönz-Bruess 2004)<br>6.6-11.8 m^3ha^{-1} downed dead wood (Ekbom et al. 2006).<br>1.7 m^3ha^{-1} dead wood in managed	5 m^3ha^{-1} each of four combinations of size and decay stage of dead wood(Stokland 2001)<br>14-18 m^3ha^{-1} snags over an area of 100 ha (Bütler et al. 2004a, b)<br>34% old forest of a local landscape (Angelstam 2004)<br>8–17 m^3ha^{-1} of standing deciduous dead trees over an area of 100 ha (Roberge et al. 2008)<br>>100-200 standing dead trees per ha (Lohmus et al. (2010))<br>Five species never recorded wherever <23.8–28.5 m^3ha^{-1} of total dead wood over an area of 100-400 ha (Økland et al. (1996))<br>Number of saproxylic species increases with increasing dead wood amounts even in the upper range of dead wood volumes studied (> 60 m^3ha^{-1}) Martikainen et al. (2000)<br>No threatened species found in managed stands with < 20 m^3ha^{-1} of dead wood; large number and high abundance of threatened species only where > 100 m^3ha^{-1} (Penttilä et al. 2004)	45-62% old-growth forest (Bélisle et al. 2011:900);<br>22-96% old-growth forest (Angelstam and Kuuluvainen 2004:125)<br>44% of state forest in boreal Sweden >150 years in 1915 (excluding mountain forest) (Linder and Östlund 1998).<br>29-57% older forest stands (>120-150 years) in three study landscapes in 1916-1921 (Linder and Östlund 1998)<br>59-67% deciduous trees in landscape (Angelstam and Dönz-Breuss 2004:313)<br>Range of means for downed dead wood 13–117 m^3ha^{-1} in boreal old-growth spruce dominated forest; 12–79 m^3ha^{-1} in boreal old-growth pine dominated forest; 16–80 m^3ha^{-1} in boreal post-fire successional forest (Siitonen 2001).<br>48.7 (range 21.9–84.9) m^3ha^{-1} downed dead wood (Rouvinen et al. 2005).<br>16-79 m^3ha^{-1} downed dead wood in unmanaged boreal forest in northern Finland and NW Russia (Aakala 2010)<br>20-51 m^3ha^{-1} downed dead wood depending on landscape, Finland and Russia (Rouvinen

productive forest across Sweden (Fridman and Walheim 2000).<br>0.1-0.4 m^3ha^{-1} downed dead wood (Bütler et al. 2004a)<br>1.2-4.3 m^3ha^{-1} downed dead wood (Ekbom et al. 2006).<br>1-4 m^3ha^{-1} downed dead wood (Similä et al. 2003)<br>0.5-1 m^3ha^{-1} snags in managed boreal forest since WWII (Linder and Östlund 1998).<br>0.3±1.0 m^3ha^{-1} dead wood on clearcuts subjected to forest fuel harvesting in boreal Finland (Eräjää et al. 2010).<br>0.7±2.1 m^3ha^{-1} dead wood on clearcuts without forest fuel harvesting in boreal Finland (Eräjää et al. 2010).		et al. 2002)<br>35.3 (range 8.0–96.3) m^3ha^{-1} dead wood (Rouvinen et al. 2005).<br>11-13 m^3ha^{-1} snags in unexploited mature boreal forest (Linder and Östlund 1992; Linder and Östlund 1998)<br>3-78 m^3ha^{-1} downed dead wood in unmanaged boreal forest in northern Finland and NW Russia (Aakala 2010)<br>23.6-47.3 m^3ha^{-1} downed dead wood depending on landscape, Finland and Russia (Rouvinen et al. 2002)<br>Standing wood 10.1-47.6 m^3ha^{-1} (Rouvinen et al. 2002)<br>12-29 m^3ha^{-1} downed dead wood (Köster et al. 2005)<br>Mean downed dead wood 90 m^3ha^{-1} in long established montane and 38 m^3/ha in long established beech forests (Christensen et al. 2005b)<br>Snags in old-growth reserves beech-fir forest of a nationalpark 1-9 mean 4.8 m^3ha^{-1} (Kanold et al. 2009)<br>Snags in spruce beech forests core zone nationalpark with bark beetle disturbance 0-12 mean 1.7 m^3ha^{-1} (Kanold et al. 2009)<br>Snags in lowland beech forests old-growth reserves 0-14 mean 5.8 m^3ha^{-1} (Müller 2005)

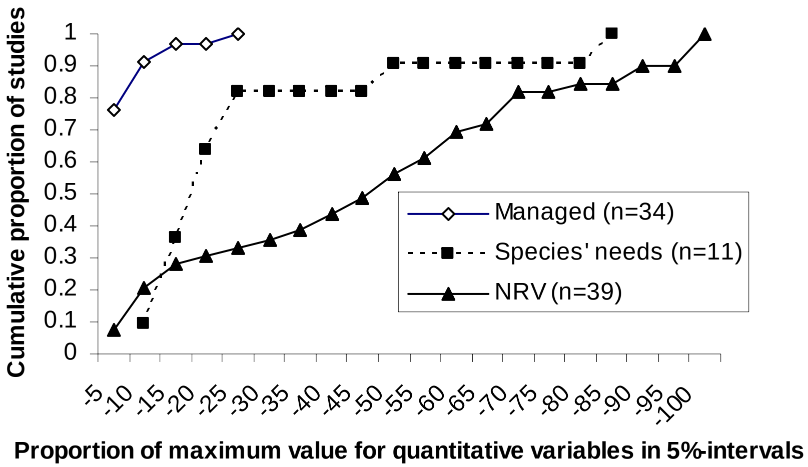

Fig. S2 Summary of results from the review of terrestrial ecosystem components (old forest, downed dead wood, standing dead wood) representing estimates about the variability in naturally dynamic forests and managed forests, and evidence-based knowledge about the requirements of specialized species of conservation concern (data sufficient only for downed dead wood) see Table 1).

References

Aakala, T. 2010. Coarse woody debris in late-successional Picea abies forests in northern Europe: Variability in quantities and models of decay class dynamics. *Forest Ecology and Management* 260: 770-779.

Angelstam, P. 2004. Habitat thresholds and effects of forest landscape change on the distribution and abundance of black grouse and capercaillie. *Ecological Bulletins* 51: 173-187.

Angelstam, P., and L. Andersson. 2001. Estimates of the needs for forest reserves in Sweden. *Scandinavian Journal of Forest Research* Supplement No. 3: 38-51.

Angelstam, P., and M. Dönz-Breuss. 2004. Measuring forest biodiversity at the stand scale – an evaluation of indicators in European forest history gradients. *Ecological Bulletins* 51: 305-332.

Angelstam, P., M. Dönz-Breuss, and J.-M. Roberge. 2004. Targets and tools for the maintenance of forest biodiversity – an introduction. *Ecological Bulletins* 51: 11-24.

Angelstam, P., and T. Kuuluvainen. 2004. Boreal forest disturbance regimes, successional dynamics and landscape structures – a European perspective. *Ecological Bulletins* 51: 117-136.

Bélisle, A.C., S. Gauthier, D. Cyr, Y. Bergeron, and H. Morin. 2011. Fires regime and old-growth boreal forest in central Quebec: an ecosystem management perspective. *Silva Fennica* 45(5): 889-908.

Bütler, R., P. Angelstam, P. Ekelund, and R. Schlaepfer. 2004a. Dead wood threshold values for the three-toed woodpecker in boreal and sub-Alpine forest. *Biological Conservation* 119: 305-318.

Bütler, R., P. Angelstam, and R. Schlaepfer. 2004b. Quantitative snag targets for the three-toed woodpecker Picoides tridactylus. *Ecological Bulletins* 51: 219-232.

Christensen, M., K. Hahn, E.P. Mountford, P. Ódor, T. Standóvar, D. Rozenbergar, J. Diaci, S. Wijdeven, et al. 2005. Dead wood in European beech (Fagus sylvatica) forest reserves. *Forest Ecology and Management* 210:267-282.

Ekbom, B., L.M. Schroeder, and S. Larsson. 2006. Stand specific occurrence of coarse woody debris in a managed boreal forest landscape in central Sweden. *Forest Ecology and Management* 221:2-12.

Eräjää, S., P. Halme, J.S. Kotiaho, A. Markkanen, and T. Toivanen. 2010. The volume and composition of dead wood on traditional and forest fuel harvested clear-cuts. *Silva Fennica* 44: 203-211.

Franc, N., F. Götmark, B. Økland, B. Nordén, and H. Paltto. 2007. Factors and scales potentially important for wood-living beetles in temperate mixed oak forests. *Biological conservation* 135: 86-98.

Fridman, J., and M. Walheim. 2000. Amount, structure, and dynamics of dead wood on managed forestland in Sweden. *Forest Ecology and Management* 131(1-3): 23–36.

Kanold, A., N. Rohrmann, and J. Müller. 2009. Einflussfaktoren auf das Baumhöhlenangebot und dessen Auswirkungen auf die Arten und Dichten von Höhlenbrütern in Bergwäldern [Factors affecting the supply of nest holes and its effects on the species and densities of hole-nesters in mountain forests]. *Ornithologischer Anzeiger* 47: 116-129 (in German).

Köster, K., K. Jõgiste, H. Tukia, M. Niklasson, and T. Möls. 2005. Variation and ecological characteristics of coarse woody debris in Lahemaa and Karula National Parks, Estonia. *Scandinavian Journal of Forest Research* 20:102-111.

Linder, P., and L. Östlund. 1992. Changes in the boreal forests of Sweden 1870-1991. *Svensk Botanisk Tidskrift* 86: 199-215.

Linder, P., and L. Östlund. 1998. Structural changes in three mid-boreal Swedish forestlandscapes, 1885–1996. *Biological Conservation* 85:9–19.

Lohmus A, M. Kinks, and M. Soon. 2010. The Importance of dead-wood supply for woodpeckers in Estonia. *Baltic Forestry* 16: 76-86.
Martikainen, P., J. Siitonen, P. Punttila, L. Kaila, and J. Rauh. 2000. Species richness of Coleoptera in mature managed and old-growth boreal forests in southern Finland. *Biological Conservation* 94:199-209.
Mikusinski, G., P. Angelstam, and U. Sporrong. 2003. Distribution of deciduous stands in villages located in coniferous forest landscapes in Sweden *Ambio* 33(8): 520-526.
Müller, J., 2005. *Waldstrukturen als Steuergröße für Artengemeinschaften in kollinen bis submontanen Buchenwäldern*. Wissenschaftszentrum Weihenstephan für Ernährung, Landnutzung und Umwelt. München: Technische Universität [Forest structure as a control variable for species communities in colline to submontane beech forests] (in German).
Økland, B., A. Bakke, S. Hågvar, and T. Kvamme. 1996. What factors influence the diversity of saproxylic beetles? A multiscaled study from a spruce forest in southern Norway. *Biodiversity and Conservation* 5: 75-100.
Penttilä, R., J. Siitonen, and M. Kuusinen. 2004. Polypore diversity in managed and old-growth boreal Picea abies forests in southern Finland. *Biological Conservation* 117: 271-283.
Roberge, J.M., P. Angelstam, and M.A. Villard. 2008. Specialised woodpeckers and naturalness in hemiboreal forest – deriving quantitative targets for conservation planning. *Biological Conservation* 141: 997–1012.
Rouvinen, S., and J. Kouki. 2008. The natural northern European boreal forests: unifying the concepts, terminologies, and their application. *Silva Fennica* 42(1): 135–146.
Rouvinen, S., T. Kuuluvainen, and L. Karjalainen. 2002. Coarse woody debris in old Pinus sylvestris dominated forests along a geographic and human impact gradient in boreal Fennoscandia. *Canadian Journal of Forest Research* 32: 2184–2200.
Rouvinen, S., A. Rautiainen, and J. Kouki. 2005. A relation between historical forest use and current dead woody material in a boreal protected old-growth forest in Finland. *Silva Fennica* 39:21-36.
Siitonen, J. 2001. Forest management, coarse woody debris and saproxylic organisms: Fennoscandian boreal forest as an example. *Ecological Bulletins* 49:11-41.
Similä, M., J. Kouki, and P. Martikainen. 2003. Saproxylic beetles in managed and seminatural scots pine forests: quality of dead wood matters. *Forest Ecology and Management* 174: 365-381.
Stokland, J.N. 2001. The coarse woody debris profile: an archive of recent forest history and an important biodiversity indicator. *Ecological Bulletins* 49: 71-83.
